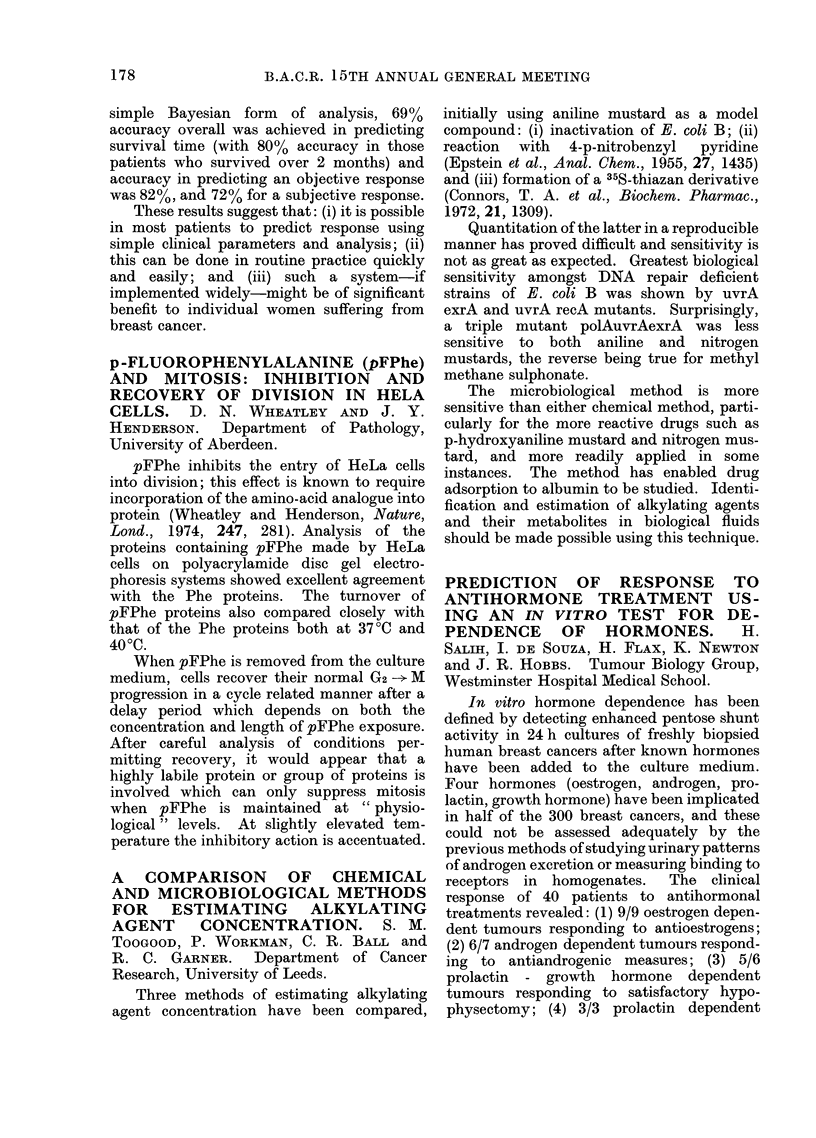# Proceedings: A comparison of chemical and microbiological methods for estimating alkylating agent concentration.

**DOI:** 10.1038/bjc.1974.149

**Published:** 1974-08

**Authors:** S. M. Toogood, P. Workman, C. R. Ball, R. C. Garner


**A COMPARISON OF CHEMICAL AND MICROBIOLOGICAL METHODS FOR ESTIMATING ALKYLATING AGENT CONCENTRATION.** S. M. TOOGOOD, P. WORKMAN, C. R. BALL and R. C. GARNER. Department of Cancer Research, University of Leeds.

Three methods of estimating alkylating agent concentration have been compared, initially using aniline mustard as a model compound: (i) inactivation of *E. coli* B; (ii) reaction with 4-p-nitrobenzyl pyridine (Epstein *et al.*, *Anal. Chem.*, 1955, **27**, 1435) and (iii) formation of a $^{35}$S-thiazan derivative (Connors, T. A. *et al.*, *Biochem. Pharmac.*, 1972, **21**, 1309).

Quantitation of the latter in a reproducible manner has proved difficult and sensitivity is not as great as expected. Greatest biological sensitivity amongst DNA repair deficient strains of *E. coli* B was shown by uvrA exrA and uvrA recA mutants. Surprisingly, a triple mutant polAuvrAexrA was less sensitive to both aniline and nitrogen mustards, the reverse being true for methyl methane sulphonate.

The microbiological method is more sensitive than either chemical method, particularly for the more reactive drugs such as p-hydroxyaniline mustard and nitrogen mustard, and more readily applied in some instances. The method has enabled drug adsorption to albumin to be studied. Identification and estimation of alkylating agents and their metabolites in biological fluids should be made possible using this technique.